# Measuring progress in global health

**DOI:** 10.1093/trstmh/tru125

**Published:** 2014-01-01

**Authors:** Simon I. Hay, Gerri McHugh

**Affiliations:** a Spatial Ecology and Epidemiology Group, Department of Zoology, University of Oxford, South Parks Road, Oxford, OX1 3PS, UK; b Royal Society of Tropical Medicine and Hygiene, Northumberland House, 303–306 High Holborn, London, WC1V 7JZ, UK

It is a pleasure to write the editorial for this issue of *Transactions of the Royal Society of Tropical Medicine & Hygiene*. This special issue accompanies the now biennial, 3-day meeting of the Royal Society of Tropical Medicine and Hygiene (RSTMH), which will take place at Oxford Town Hall (St Aldate's, Oxford, OX1 1BX) from 25–27 September 2014.

On assuming office, one of the first presidential duties^1^ is to set the theme for the biennial conference with the Chief Executive and to seek approval of the Trustees. We quickly chose the theme ‘Measuring Progress’ for the 2014 meeting and will elaborate here the reasons for that choice.^2^ Given the lead time in preparing for the conference, our deliberations were being held at the time of the publication of the 2013 annual letter of the Bill & Melinda Gates Foundation titled, ‘Why Measurement Matters.’^3^ The ideas in the annual letter were, in part, catalysed by William Rosen's thesis,^4^ that it was improvement in and use of precise measurement tools, which allowed the innovations necessary to efficiently harness steam power and thus spur the industrial revolution. This idea, along with insights gained leading various activities of the Bill & Melinda Gates Foundation, were combined to provide a compelling argument for setting clear goals in global public health and determining the mechanisms to measure progress towards them. The idea resonated with the current President, an epidemiologist long-interested in measuring progress in reducing the burden of a range of infectious diseases, mostly by cartographic methods.^5–8^ The ideas also resonated with the RSTMH leadership. It was clear that measuring progress was a theme that could embrace the full diversity of our fellowships' interests (past, present and, importantly, future) and thus be of wide appeal. The idea was duly adopted and is duly attributed here.

Over our 3 days in Oxford, therefore, the focus will be on how to measure progress in our diverse global health activities to ensure we are having maximum impact. To help the RSTMH in this objective we have been fortunate to attract a distinguished array of keynote speakers and session chairs (more details below), who have helped shape the scientific content of the sessions around the theme of measuring progress. The full details of the programme are available on the conference website (https://rstmhmeasuringprogress.org/scientific-programme) and include representatives of the development aid community, the charitable sector, international funding agencies, as well as the more usual suspects from academia.

All conference participants have been encouraged to translate the measuring progress theme into their own working areas. The final selection of these working areas are 1. The global burden of disease: measuring morbidity and mortality at finer temporal and spatial scales, chaired by Professor Chris Murray, Institute for Health Metrics and Evaluation; 2. Resistance: measuring resistance to insecticides and drugs, and its threat to progress, chaired by Professor Janet Hemingway, Liverpool School of Tropical Medicine; 3. Sexually transmitted diseases: measuring progress in control, chaired by Professor Peter Piot, London School of Hygiene and Tropical Medicine; 4. Development aid: measuring progress achieved with international financing, chaired by Professor Chris Whitty, Department for International Development; 5. Neglected tropical diseases: measuring progress towards 2020 control and elimination goals, chaired by Professor Don Bundy, The World Bank, and Julie Jacobson, Bill & Melinda Gates Foundation; 6. Trials: methods for measuring progress and impact, chaired by Professor Helen McShane, University of Oxford; 7. Charitable sector: strategies for measuring progress, chaired by Dorothy Dalton, *Editor of Governance*, and; 8. Animal health and zoonoses: measuring progress in One Health, chaired by Professor James Wood, University of Cambridge.

We are delighted to announce that Her Royal Highness, The Princess Royal, Honorary Fellow of the RSTMH, will be supporting the themes of the conference by attending sessions on the morning of Friday 26 September. We remain extremely grateful for The Princess Royal's long-standing support of the Society's work and very much look forward to welcoming Her Royal Highness to Measuring Progress. We also extend our warm welcome and thanks to Alan Magill, in his capacity as President of the American Society of Tropical Medicine and Hygiene, and Jeremy Farrar, as Director of the Wellcome Trust, as our distinguished keynote speakers. Chris Murray will also be giving the RSTMH Chadwick Memorial Prize Lecture, ‘Measuring progress in malaria: challenges and options for the future,’ in the Sheldonian Theatre on the evening of Wednesday 24 September. This re-established prize lecture will now be given at each biennial in memory of Sir Edwin Chadwick KCB (1800–1890), an English social reformer who did a huge amount to improve sanitary conditions and public health in Britain.^9^

The RSTMH strives to continually develop communications with our fellowship so we will be streaming live as much of the conference content as possible so that those unable to travel to Oxford can participate. In addition, we are extremely grateful for the generous support from the Bill & Melinda Gates Foundation and the Wellcome Trust that has enabled us to offer nearly 30 travel scholarships to early-career investigators from across the developing world to attend and present at Measuring Progress.

This editorial similarly serves as a valuable communication tool with the fellowship. We use it to highlight a collection of three commissioned commentaries and a further three reviews that reflect many of the themes of the Measuring Progress conference.

Hill and Whalen start the commentaries by reflecting on the importance of socio-economic factors associated with TB incidence changes and how these complicate measuring progress of DOTS (Directly Observed Treatment, Short-course).^10^ Dalton then comments on the current crisis in human resources for health in Africa.^11^ The focus is on well-documented success stories on how to build a sustainable workforce and thereby provide insights on how progress can be maximised by learning from these examples. Deribe and Davey conclude the commentaries by looking back,^12^ in this case to an article published in *Transactions of the Royal Society of Tropical Medicine & Hygiene* by Price in 1976: ‘The association of endemic elephantiasis of the lower legs in East Africa with soil derived from volcanic rocks.’^13^ They show how this article has defined progress in podoconiosis research and, moreover, 30 years after publication, progress can be measured by the use of historical data to understand trends in disease distribution. This commentary reflects an area in which we are very keen to commission contributions; tracing the impact of influential articles in the *Transactions of the Royal Society of Tropical Medicine & Hygiene* archives. Articles have been published continually since 1907 and are freely, available electronically to the fellowship.

Gutiérrez starts the review section by looking at factors that have contributed to reducing the impact of snakebite envenoming in Latin America and the Caribbean in the last decade and challenges for measuring progress in the next decade.^14^ Babu and Babu continue the measuring progress theme by systematically reviewing coverage and compliance with mass drug administration under the programme to eliminate lymphatic filariasis in India.^15^ Gaps between coverage and compliance are documented, as well as correctable causes, which need to be addressed if the Indian programme is to measure progress towards elimination of lymphatic filariasis. Mnzava et al. pick up many of the themes of the conference by arguing that global reductions in malaria burden could be undermined by a lack of field-orientated vector biologists with skills to manage insecticide resistance, monitor intervention coverage and identify areas of residual transmission and that measures should be adopted to address this shortage.^16^

We hope that the conference (in person or online) and this editorial will go some way to promoting the measuring progress theme among our fellowship. We further hope to document the enduring impact of this measuring progress theme on your work in future contributions to our flagship journals.

The Measuring Progress papers in this issue, along with papers from our sister journal, *International Health*, will be available in a free online Measuring Progress collection until the end of October 2014 at http://trstmh.oxfordjournals.org/.
